# Mutation in Exon2 of *BRCA1* Gene in Adult Bengali Bangladeshi Female Patients with Breast Cancer: An Experience from Two Tertiary-Care Hospitals

**DOI:** 10.31557/APJCP.2020.21.8.2265

**Published:** 2020-08

**Authors:** Sagana Shahreen Chowdhury, Marjia Khatun, Toufiq Hasan Khan, Anjuman Banu Laila

**Affiliations:** *Department of Anatomy, Bangabandhu Sheikh Mujib Medical University, Shahbag, Dhaka, Bangladesh. *

**Keywords:** Breast cancer, exon2 of BRCA1 gene, nonsynonymous and synonymous mutation, c.68-69delAG

## Abstract

**Background:**

The occurrence rate of *BRCA1* mutations is found to be high in South Asian countries where early onset of breast cancer is common. In Bangladesh, noticeable percentage of patients experience breast cancer in their reproductive ages. The objective of this study was to identify any mutation in exon2 of the *BRCA1* gene in adult Bengali Bangladeshi female patients with breast cancer.

**Methods:**

In this cross-sectional descriptive study, the genomic DNA was extracted from the blood of adult fifty Bengali Bangladeshi female breast cancer patients. The whole region of exon2 of the *BRCA1 *gene was amplified and the amplified DNA products were sequenced using Sanger sequencing. The raw chromatogram data were analyzed using Chromas software, and analyzed sequences were compared with the NCBI RefSeq database by BLAST search. The resultant amino acid change was detected by MEGA X software.

**Results:**

We found the mean age at diagnosis 44.66 years, whereas 96% of patients were married, 90% were multiparous and 86% breastfed their children. All patients had unilateral breast cancer and among them 94% had invasive ductal carcinoma. Only 24.5% of the patients had associated omorbidity. The family history of breast cancer or other BRCA-associated cancer was positive only for 4% of patients. A total of five mutations were identified all of which caused by substitutions. Among them three were nonsynonymous and two were synonymous. Only 2.5% of the patients, within the age group of 18-50 years, were found to have mutations in their blood, whereas 26.66% of the patients above 50 years found to have mutations in this study.

**Conclusions:**

Among this small sample size, we found five mutations in exon2 of the *BRCA1* gene and this indicates the necessity to find out the mutation spectra of the *BRCA1* gene in the Bangladeshi population.

## Introduction

Breast cancer is the most common cancer of women worldwide. The Global Cancer Project provides evidence that 20,88,849 (11.6% of all cancers) new cases of breast cancer were identified around the world in the year 2018 and 6,26,679 deaths (6.6% of all cancer deaths) occurred in the same year (Globocan, 2018). It is predicted that the new cases of breast cancer patients will reach up to 22 million in next two decades (Ghoncheh et al., 2016). Like Western countries, it is now growing strongly in Asia and has become the leading cause of death among women (Story et al., 2011).

Many human diseases, including breast cancer, are caused by genetic defect and in the recent years, researchers and scientists are investigating the genetic cause for understanding the course of these diseases as well as for finding the way of their prevention and cure. *BRCA1* and *BRCA2* genes are found to be strongly associated with breast cancer and the frequency of these genetic mutations varies among ethnic groups and countries (Kim and Choi, 2013). Approximately 10-15% of breast cancer cases are caused by hereditary genetic mutation of *BRCA1* and *BRCA2* genes (Bhashkaran et al., 2019). Such mutations are associated with lifetime risk of breast cancer which is approximately 70% by age 80 years (Mehemmai et al., 2019).

Both *BRCA* genes are tumor suppressor genes that are responsible for tissue specific, clinically important tumor suppression (National Center for Biotechnology Information [NCBI], 2018). BRCA1 breast cancer susceptibility protein is used by the cell in an enzymatic pathway which repairs DNA molecules that have double stranded breaks (National Center for Biotechnology Information [NCBI], 2018). Besides DNA repair, it is involved in cell cycle control and regulation of apoptosis (Hartwig et al., 2013). Exon2 is one of the most common mutation sites in *BRCA1* gene for c.68-69delAG (BIC: 185delAG) frameshift mutation which occurs in codon 23 of exon2, that results in the creation of stop codon which leads to premature termination of translation and significant truncation of protein, and is associated with the severity of the disease like early onset, bilaterality of breast cancer (Hartwig et al., 2013; Chakraborty et al., 2015). The majority of breast cancers occur before age fifty (50) in most South Asian countries and it is supposed to cause by protein truncating mutations (nonsense mutation) in *BRCA* (Hopper et al., 1999; Liede and Narod 2002). The young age group (pre-menopausal) had more frequency of breast cancer in the Indian population (Shah et al., 2018). According to NICRH (National Institute of Cancer Research and Hospital) of Bangladesh, the mean age of the breast cancer patients in Bangladesh is 41.8 years and over 56% of the cases are women of reproductive age (Hossain et al., 2014). The incidence rate of *BRCA1* mutation is found higher in India and Pakistan than that of *BRCA2* mutation (Shah et al., 2018). Being the part of this subcontinent, higher prevalence of *BRCA1* mutation could be assumed for Bangladesh. Very limited studies, based on *BRCA *mutations, are found on the Bangladeshi population. Although identification of *BRCA1* and *BRCA2 *has greatly increased the understanding in breast cancer genetics in Western populations but the role of these genes for developing breast cancer in the Bangladeshi population still remains unexplored. As because the incidence of breast cancer is also rising in Bangladesh day by day, it is now demand of time to find out the genetic basis of breast cancer on Bangladeshi perspective.

## Materials and Methods

The cross-sectional descriptive study was carried out in the Genomic Research Laboratory of the Department of Anatomy, Bangabandhu Sheikh Mujib Medical University (BSMMU), and Dhaka from March 2019 to February 2020 after getting formal approval from the Institutional Review Board (IRB) of BSMMU. A total fifty (50) adult Bengali Bangladeshi (mainstream Bangladeshis originate from Bengali ethnic group) female breast cancer patients were selected using selection check list from the Department of Oncology, Bangabandhu Sheikh Mujib Medical University (BSMMU) and from the Department of Radiotherapy, Dhaka Medical College Hospital on the basis of exclusion and inclusion criteria after taking permission through the proper channel. The sociodemographic, reproductive, disease condition-related characteristics of the patients were collected using a data collection sheet.

*Isolation of DNA*

Genomic DNA was extracted from the blood sample by using commercial DNA extraction kit ReliaPrep™ (Promega, USA) as per manufacturer’s instructions. Two hundred (200) µl blood contained in the microcentrifuge tube was mixed with proteinase K and cell lysis buffer. Binding buffer, wash buffer and elution buffer were used sequentially for yielding the extracted DNA. The amount of the reagents was taken according to the SOP (Standard Operating Procedure) of DNA extraction procedure from blood. The quantity and quality of the extracted DNA were measured and checked by NanoDrop spectrophotometer. 

*Amplification*

**Figure 1:**
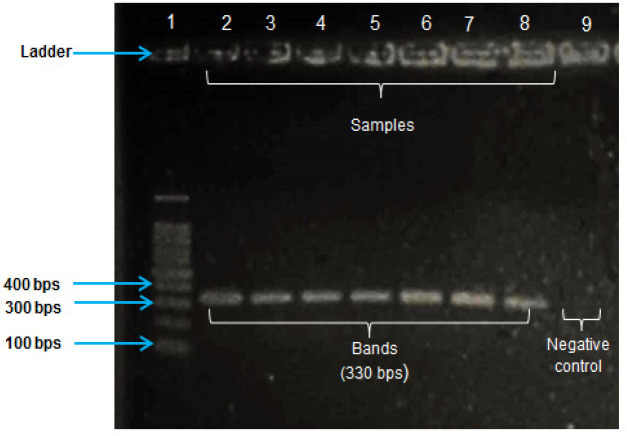
Gel Electrophoresis of the PCR Products on 1% Agarose Gel. The desired product size (330 bps) of the amplicons is shown in specific bands from lane 2 to 8 in comparison with standard 100 bps DNA ladder in lane 1. The well of lane 9 was loaded with the sample of negative control which shows no band indicating absence of contamination

Primers for exon2 of *BRCA1* gene were designed by using Primer3Plus software. Exon2 (target region) has got 99 bps nucleotides. Both primers (forward primer and reverse primer) were designed such a way that they included both flanking regions (at 5ˊ end and 3ˊ end) of exon2 of *BRCA1* gene (Table 1). The desired portion of *BRCA1* gene was amplified by conventional PCR. Amplification of this sequence was accomplished by short-range PCR. Amplification was performed on a Biometra thermal cycler (Biometra GmbH, Germany). The amplicons were visually confirmed by 1% agarose gel electrophoresis (Figure 1). PCR products were sent for Sanger sequencing.

*DNA sequencing*

For Sanger sequencing, the PCR products were run by using ABI-3500 Genetic Analyzer (Thermo Fisher Scientific, USA). For running the cycles, BigDye® Terminator v3.1 Cycle Sequencing Kit was used according to the standard protocol provided with it. 

*Data analysis*

Sanger sequencers generated a four-color chromatogram which represented the results of the sequencing run. For the present research, data obtained from the sequence in ABI files were analyzed using the Chromas® software. The sequences were compared with the NCBI (National Centre for Biotechnology Information) database by BLAST search. Automatic translations of codons were explored by MEGA X software. The translated codons were compared with the reference sequence obtained from NCBI Entrez search engine. The percentage frequency of mutations among the socio-demographic, reproductive and disease condition-related characteristics of the breast cancer patients were analyzed by SPSS (Statistical Package for the Social Sciences,version 24).

*Ethical implication*

In this study, all breast cancer patients were treated equally and with respect. The aim and possible benefits of the study were explained to the respondents who participated in the study. Verbal and written consents were obtained from all the breast cancer patients. Each patient was given a special ID number for safeguarding confidentiality and protecting anonymity. All selected patients were informed that their DNA samples were used for research purpose only. They also were informed that they had the right to refuse to participate in or withdraw their names from the study at any time. 

## Results

*Mutation results*

**Figure 2:**
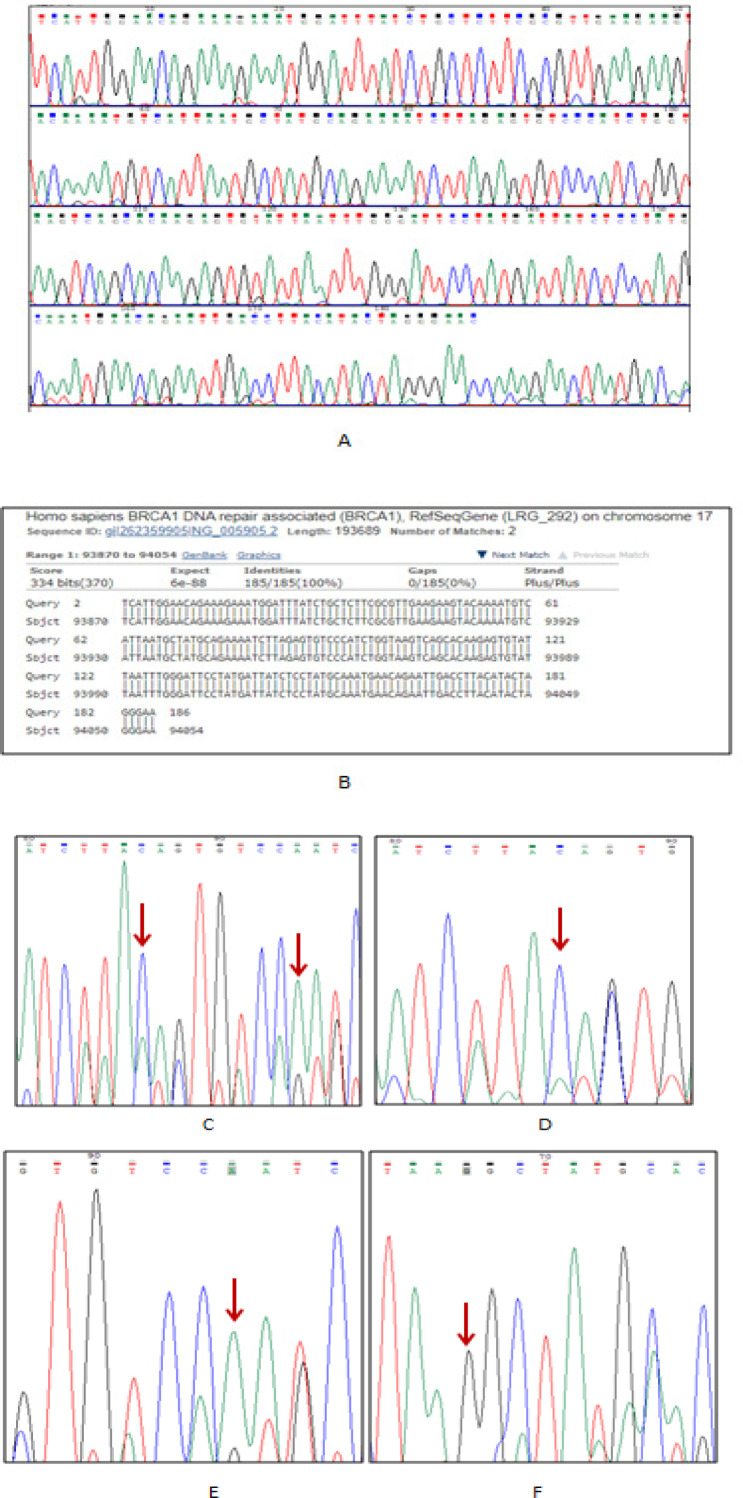
Sequence of Sample ID- BC 30 Using as the Control Chromatogram Sequence (A) after 100% matching with the BLAST searches (B). In picture ‘C’, at 86th nucleotide position (67th position of coding sequence), ‘G’ has been substituted by ‘C’ (c.67G>C) and at the 94th nucleotide position (75th position of coding sequence), ‘C’ has been substituted by ‘A’ (c.75C>A) in sample ID- BC 5 as shown by the red arrow. In picture ‘D’, at 86th nucleotide position (67th position of coding sequence), ‘G’ has been substituted by ‘C’ (c.67G>C) in sample ID- BC 6 (red arrow). In picture ‘E’, at the 94th nucleotide position (75th position of coding sequence), ‘C’ has been substituted by ‘A’ (c.75C>A) in sample ID- BC 10 (red arrow). In picture ‘F’, at the 67th nucleotide position (48th position of coding sequence), ‘T’ has been substituted by ‘G’ (c.48T>G) in sample ID- BC 4 (red arrow)

A total of five mutations were identified, all of which caused by substitutions. The identified mutations were c.48T>G (p.Asn16Lys), c.67G>C (p.Glu23Gln) and *c.75C>A (p.Pro25Pro)*. Among these mutations, two mutations, identified in one patient, namely c.*67G>C* (p.Glu23Gln) where ‘G’ was substituted by ‘C’ and *c.75C>A (p.Pro25Pro)* where ‘C’ was substituted by ‘A’, of which the first one was nonsynonymous (missense) and other was synonymous (silent) mutation. The very same mutations were detected in two other patients. Another nonsynonymous mutation, *c.48T>G (p.Asn16Lys)*, was detected where ‘T’ was substituted by ‘G’ at the 48th nucleotide position of coding sequence. All are presented in Figure 2. All the chromatogram sequences were compared with a control sequence which was matched with the reference sequence of exon2 of *BRCA1* gene by BLAST search (Figure 2). In this study, the nucleotide and protein sequence data of identified mutation namely *c.75C>A (p.Pro25Pro)* had been submitted to GenBank for accession number, as it was not reported in any existing databases but observed by an Indian author in the Indian population, through a web-based sequence submission tool ‘BankIt’. The mutation report is given in Table 2. 

*Sociodemography of the patients*

The mean age at diagnosis of the adult female breast cancer patients was 44.66 years, and 96% of patients were married with no family history of breast cancer among the 1^st ^degree relatives. The mean ages at menarche and menopause were 12.06 years and 47.04 years respectively. About 90% of patients were multiparous and 86% of patients breast fed their children. About 42% of patients never used any contraceptive. All the patients had unilateral breast cancer whereas 94% of patients were diagnosed as invasive ductal carcinoma. Only 24.5% patients had associated comorbidities like diabetes mellitus, hypertension or ischemic heart disease.

*Relationships of the identified mutations with the characteristics*

Among the fifty breast cancer patients, four mutations (26.66%) were found in the age at diagnosis above fifty. Only one patient (2.85%) had mutation at the age at diagnosis between 18 and 50. All the breast cancer patients, identified of having mutations, were married and multiparous. The mutation rate was higher in the married patients (10.41%) than in the unmarried patients (0%), and very similar findings were noted in case of multiparous patients (11.11%) in comparison to the nulliparous patients (0%). Regarding menstrual status, higher percentage of postmenopausal breast cancer patients (16%) had mutations than premenopausal breast cancer patients (4.1%). Mutations were present in 14.28% of breast cancer patients who used oral contraceptives, in 11.62% of patients who breast fed their babies for more than 6 months. About 10.41% of patients, who had no family history of having breast cancer or other *BRCA1*-related cancers among their 1^st^-degree relatives, and about 33.33% of patients, who had comorbidities, were found to have mutations. Regarding disease condition-related characteristics, all the five mutations were present among the patients who had left-sided breast cancer and had been suffering from invasive type of ductal carcinoma. 

**Table 1:** The Sequences of Oligonucleotide Primers and Their Relevant Information

Primer	Sequence	Length	GC %	Tm (ºC)	Product size
F	5ˊGGACGTTGTCATTAGTTCTTTGGT 3ˊ	24	41.7	60.7	330 bps
R	5ˊTCCCTAGTATGTAAGGTCAATTCTG 3ˊ	25	40	57.9	

Forward primer, F. Reverse primer, R

**Table 2:** Report of the Identified Mutations with Reference Numbers

Nucleotide position	DNA reference number	Protein reference number	Clinical significance
c.48T>G	NM_007298.3:c.48T>G	NP_009229.2:p.Asn16Lys	Uncertain significance
c.67G>C	NM_007294.3:c.67G>C	NP_009225.1:p.Glu23Gln	Uncertain significance
c.75C>A	MN968555	Not recorded	Not recorded

## Discussion

We found 5 mutations in exon2 of *BRCA1* gene from blood of fifty adult female breast cancer patients. *BRCA1* gene has 300 disease causing mutations like small insertion, small deletion, single nucleotide substitution, splicing trouble, nonsense mutation, missense mutation (Mehrgou and Akouchekian, 2016). The Most of the breast cancer-causing mutations in *BRCA1* gene lead to produce truncated protein through the nonsense, frame shift and splicing mutations (Mehrgou and Akouchekian, 2016). But, we found no protein truncating mutation in our study. All the mutations were caused by substitutions of which three were nonsynonymous mutations and two were synonymous mutations.

The mean age at diagnosis of our study population was 44.66 years. Our result regarding the mean age at diagnosis supports the other studies conducted on the Bangladeshi population which is also close to the study populations of the neighbor countries like India (45-50 years) and Pakistan (48.6 years), and also South East Asian countries (45-50 years) (Leong et al., 2010; Hossain et al., 2014; Badar et al., 2015; Nishat et al., 2019). But in United States it is 70 years and in Europe it is 67 years (Abdulrahman et al., 2012). From earlier studies, we can say that the mean age at diagnosis of the breast cancer patients in Asia is still substantially younger than that in Europe and America. In our study, we found that 70% patients had developed their breast cancers in their forties.

The frequency of breast cancer among the married women was found higher (96%) than that of the unmarried women which is consistent with the findings (93%) of other studies conducted on the Bangladeshi population (Hossain et al., 2014; Nishat et al., 2019). A possible explanation could be that most of the women in Bangladesh have experienced early marriage. A study shows that the mean age of first marriage is 18.7 years for Bengali Bangladeshi women, whereas 93% breast cancer patients were married (Hossain et al., 2014). A study in India shows nearly similar results regarding marital status where breast cancer has been found in 84.41% married women (Chakrabortyet al., 2015). In America, 42.6% breast cancer patients were unmarried during their diagnosis (Martinez et al., 2016). In many European and American countries, marriage rates have been declined which is accompanied with the increased average age of getting married as well as increased the rates of divorce (OECD Family Database, 2019).

The risk of breast cancer has been increased with the early age of menarche and late age of menopause due to the longer period exposure to ovarian sex steroids observed by many previous studies. The mean age at menopause in Indian women is 46.2 years and in Pakistani women is 49.2 years which is consistent with the finding of the present study (47.4 years) (Ahuja, 2016; Waheed et al., 2016). But in United States, it is 52 years (Peacock and Ketvertis, 2019). Multiple birth and breast feeding for a long period are the common features in Bangladeshi society which reflects in the results of the present study where 90% of patients were multiparous and 86% breast fed their children for a period more than six months. We found, no bilateral breast cancer which is consistent with the findings of another study where no bilaterality was found (Bhattacharjee et al., 2018). In Mexico, 11% of the breast cancer patients had diabetes mellitus and 18% had hypertension whereas we found 24.5% of the breast cancer patients to have comorbidities (hypertension or/and diabetes mellitus) (Noveron et al., 2017).

185delAG (c.68_69delAG) is one of the most common founder mutations which is very common in Ashkenazi Jewish population who are suffering from early onset of breast cancer, and has also been observed in Asian, American, African and European populations including the countries such as India, Russia, Iran, non-Jews Morocco population, Netherland, Switzerland, Portugal, Slovakia, Hungary, Brazil, Lebanon, Egypt (Karami and Mehdipour, 2013). It has also been detected in Arabic ethics including Syria, Iraq and Yemen (Karami and Mehdipour, 2013). One Indian study shows the high incidence (16.3%) of c.68_69delAG mutation in Indian population which is near the frequency (18%) of this mutation in Ashkenazi Jewish (Karami and Mehdipour, 2013). Pakistan also has c.68_69delAG mutation (Liede and Narod, 2002). In addition to early onset of breast cancer, c.68_69delAG has strong involvement in familial cases (Francis et al., 2015). In the present study no such type of mutation has been found. To our knowledge, this type of mutation has not yet reported in any studies conducted on the Bangladeshi population.

In our study, the mutation rate for *BRCA1* has been found much lower (2.85%) in the patients with the age range 18-50 years than that of the patients above 50 years (26.66%). All patients having *BRCA1* mutation were married, multiparous, breast feeding women, had no family history of breast cancer among their first-degree relatives, and had unilateral invasive ductal breast cancers. Earlier studies show that mutation in *BRCA1* gene increases the risk for developing early-onset and bilateral breast cancer (Mehrgou and Akouchekian, 2016). A study conducted in North India, shows early onset of breast cancer with positive family history was associated with *BRCA1* gene mutation (Saxena et al., 2002). On the contrary, a study shows that 72.3% of participants among the *BRCA1* variant carriers did not report any family history of cancer (Bhaskaran et al., 2019). In our study, one patient with *BRCA1* gene mutation has shown the early-onset of breast cancer who’s age at diagnosis was 35 years. But contrary to the other findings, she had unilateral breast cancer and had no positive family history of breast cancer. In our study, two other patients, who had *BRCA1* mutations, were diagnosed at their fifties (55 years and 59 years), and one patient was diagnosed in her sixties (60 years). A study showed no statistically significant difference between the age at onset of breast cancer and the *BRCA* mutation, as compared with those without mutations (Wang et al., 2018).

In conclusion, among this small sample size, we found five mutations in exon2 of *BRCA1* gene from blood samples. Besides that, we observed some selected sociodemographic, reproductive and disease condition-related characteristics of the breast cancer patients which reflect some extent of epidemiological characteristics of breast cancer on Bangladeshi perspective. We also made relationships of these characteristics with the identified mutations to provide an idea about the mutational status among the study population. So, in future, if a study conducted with larger sample size, mutation detection from blood may help in enriching the database of mutational spectra of Bengali Bangladeshi female breast cancer patients.

## References

[B1] Abdulrahman GO, Rahman GA (2012). Epidemiology of breast cancer in Europe and Africa. J Cancer Epidemiol.

[B2] Ahuja M (2016). Age of menopause and determinants of menopause age: A pan India survey by IMS. J Mid-life Health.

[B3] Badar F, Faraz SMR, Yousaf A (2015). Epidemiology of breast cancer at the Shaukat Khanum Memorial Cancer Hospital and Research Center, Lahore, Pakistan. J Coll Physicians Surg Pak.

[B4] Bhaskaran SP, Chandratre K, Gupta H (2019). Germline variation in BRCA1/2 is highly ethnic-specific: Evidence from over 30,000 Chinese hereditary breast and ovarian cancer patients. Int J Cancer.

[B5] Bhattacharjee A, Hossain AFMA, Yeasmin S, Akter T (2018). Incidence, epidemiology and clinico-pathological status of different molecular subtypes of breast cancer in NICRH, Dhaka. Delta Med Col J.

[B6] Chakraborty A, Banerjee D, Basak J, Mukhopadhyay A (2015). Absence of 185delAG and 6174delT mutations among breast cancer patients of Eastern India. Asian Pac J Cancer Prev.

[B7] Francis AM, Ramya R, Nalini G (2015). Analysis of BRCA1 gene exon 2 mutation in breast cancer patients in a South Indian population. Res J Pharm Technol.

[B8] Ghoncheh M, Pournamdar Z, Salehiniya H (2016). Incidence and mortality and epidemiology of breast cancer in the world. Asian Pac J Cancer Prev.

[B10] Hartwig M, Janiszewska H, Bak A (2013). Prevalence of the BRCA1 c 68_69delAG (BIC: 185delAG) mutation in women with breast cancer from north-central Poland and a review of the literature on other regions of the country. Contemp Oncol.

[B11] Hopper JL, Southey MC, Dite GS (1999). Population-based estimate of the average age-specific cumulative risk of breast cancer for a defined set of protein truncating mutations in BRCA1 and BRCA2. Cancer Epidemiol. Biomarkers Prev.

[B12] Hossain MS, Ferdous S, Karim-Kos HE (2014). Breast cancer in South Asia: A Bangladeshi perspective. The International J Cancer Epidemiol Detect Prev.

[B13] Karami F, Mehdipour P (2013). A comprehensive focus on global spectrum of BRCA1 and BRCA2 mutations in breast cancer. Biomed Res Int.

[B14] Kim H, Choi DH (2013). Distribution of BRCA1 and BRCA2 mutations in Asian patients with breast cancer. J Breast Cancer.

[B15] Leong SPL, Shen Z, Liu T (2010). Is breast cancer the same disease in Asian and Western countries?. World J Surg.

[B16] Liede A, Narod SA (2002). Hereditary breast and ovarian cancer in Asia: Genetic epidemiology of BRCA1 and BRCA2. Hum Mutat.

[B17] Martinez ME, Anderson K, Schwab R (2016). Marital status and overall mortality in breast cancer patients: Differences by socioeconomic status and race/ethnicity. J Cancer Res.

[B18] Mehrgou A, Akouchekian M (2016). The importance of BRCA1 and BRCA2 genes mutations in breast cancer development. Med J Islam Repub Iran.

[B19] Mehemmai C, Cherbal F, Hamdi Y (2019). BRCA1 and BRCA2 germline mutation analysis in hereditary breast/ovarian cancer families from the Aures Region (Eastern Algeria): First report. Pathol Oncol Res.

[B22] Nishat L, Yesmin ZA, Arjuman F, RahmanSHZ, Banu LA (2019). Identification of mutation in exon11 of BRCA1 gene in Bangladeshi patients with breast cancer. Asian Pac J Cancer Prev.

[B23] Noveron NR, Garza CV, Celis ESP (2017). Clinical and epidemiological profile of breast cancer in Mexico: Results of the Seguro popular. J Glob Oncol.

[B26] Saxena S, Szabo CI, Chopin S (2002). BRCA1 and BRCA2 in Indian breast cancer patients. Hum Mutat.

[B27] Shah ND, Shah PS, Panchal YY (2018). Mutation analysis of BRCA1/2 mutations with special reference to polymorphic SNPs in Indian breast cancer patients. Appl Clin Genet.

[B28] Story HL, Love RR, Salim R (2011). Improving outcomes from breast cancer in a low-income country: Lessons from Bangladesh. Int J Breast Cancer.

[B29] Waheed K, Khanum A, Ejaz S, Butt A, Randhawa FA (2016). Quality of life after menopause in Pakistani women. Gynecol Obstet.

[B30] Wang YA, Jian J, Hung C (2018). Germline breast cancer susceptibility gene mutations and breast cancer outcomes. BMC Cancer.

